# Improving the Antitumor Effect of Chemotherapy with Ocoxin as a Novel Adjuvant Agent to Treat Prostate Cancer

**DOI:** 10.3390/nu15112536

**Published:** 2023-05-29

**Authors:** Iera Hernandez-Unzueta, Aitor Benedicto, Uxue Telleria, Eduardo Sanz, Joana Márquez

**Affiliations:** 1 Cell Biology and Histology Department, Medicine and Nursing Faculty, University of the Basque Country, 48940 Leioa, Spain; 2 Research and Development, Catalysis S.L., 28016 Madrid, Spain

**Keywords:** prostate cancer, cancer nutrition, adjuvant, chemotherapy, resistance, tumor microenvironment, fibroblast, osteoblast

## Abstract

Prostate cancer is one of the most common cancers among men. Although many patients respond favorably to first-line treatments, castration—and chemotherapy—resistance arises after a few years, leading to metastasis. Thus, new approaches are being investigated using natural supplements to reinforce current therapies. Ocoxin is a plant-based mixture with antitumor properties that have been proved in several cancers. Here, we evaluated the cytotoxic capacity of this compound itself and combined with Docetaxel, Enzalutamide and Olaparib as an adjuvant agent. We observed that Ocoxin reduced tumor cell viability; slowed down cell cycles; altered the expression of genes involved in DNA replication, cell cycles and the p53 signaling pathway; and reduced migratory capacity after stimulation with cancer-associated fibroblasts (CAFs) and osteoblasts in vitro and reduced tumor volume in vivo. The combination of the nutritional supplement with chemotherapy showed a higher cytotoxic effect than chemotherapy alone and reverted chemoresistance conferred by CAFs and osteoblasts. Moreover, the adjuvant therapy also improved the outcome in vivo compared to the treatment with solo chemotherapy, where mice developed smaller tumors and less angiogenesis. Therefore, Ocoxin arises as a good candidate for further studies in combination with current treatments for prostate-cancer patients.

## 1. Introduction

Cancer is one of the leading causes of death worldwide. In particular, prostate cancer is the second most common cancer among men and the fifth cause of cancer death [1]. These tumors originate from prostate cells that have undergone genetic and epigenetic changes such as an increase in oncogene expression and the inhibition of tumor suppressors, leading to an uncontrolled cell proliferation, which results in a prostate adenocarcinoma [2]. Even though there are procedures such as resection or radiotherapy for localized primary tumors, some malignant cells can spread through the bloodstream or lymphatic system and cause metastasis. In this scenario, androgen deprivation therapy (ADT) is used as the first-line treatment with drugs such as enzalutamide that block androgen receptors [3,4]. Although most patients respond favorably, 10–20% of prostate cancer cases become castrate-resistant after 2–3 years [5]; thus, additional medication such as chemotherapy or immunotherapy is administered in order to stop tumor growth. In this scenario, docetaxel is the most prescribed drug, which acts as an antimitotic agent which impedes cell division. Nevertheless, occasionally, cancer cells do not react to these agents, leading to patient death [2,6]. Recently, an inhibitor of poly-ADP-ribose polymerase (PARP) named Olaparib was approved for the treatment of prostate cancer, showing a favorable outcome [7,8]. However, this agent does not always improve patient’s survival [9] and, as well as the previously mentioned drugs, it worsens their quality of life (QoL) due to side-effects [10,11]. Therefore, innovative approaches are needed not only to cure prostate cancer but also to ameliorate the detrimental adverse reactions provoked by those therapies.

Evidence indicates that treatment failure lies partly in the interaction of tumor cells with the tumor microenvironment (TME) [12,13], which, besides cancer cells, contains many elements such as stromal cells, immune cells, extracellular matrix (ECM), cytokines, growth factors and many other extracellular components. Therefore, it is also necessary to consider TME when treating cancer. Prostate tumors usually show a reactive stroma with a remarkable cancer-associated fibroblast (CAF) and immune-cell infiltration [12,14,15]. CAFs comprise the main component of the TME and show features of activated fibroblasts found in inflammatory milieu. These cells remodel ECM by producing collagen and matrix metalloproteinases (MMPs), cause hypoxia, induce angiogenesis and secrete factors which can facilitate invasion and induce the recruitment of immune cells such as macrophages and regulatory T cells [16]. In this regard, tumor-associated macrophages (TAMs) constitute the major immune-cell type of the TME, whose infiltration can increase the secretion of pro-tumoral factors [17,18] and create an immunosuppressive environment [19], facilitating tumor development and metastasis. Interestingly, cancer cells are known to secrete IL-6, which induces the polarization of M2 macrophages [20] which, in turn, produce anti-inflammatory molecules and suppress antitumor responses [21]. Moreover, another type of immune cell hosted in the TME is lymphocytes. In particular, cytotoxic T lymphocytes (CTLs) are of special interest since they influence the anticancer response [22,23]. Therewith, several studies have confirmed that TME confers resistance to ADT, chemotherapy and also to immunotherapy [24,25,26,27,28], giving rise to tumor dissemination.

Nonetheless, while primary prostate-cancer tumors grow with a certain TME, metastatic cancer evolves in a completely different tissue. Prostate-cancer metastases occur in liver, lung and, predominantly, bones, where tumor cells secrete cytokines, chemokines, hormones, growth factors and metabolites which stimulate the maturation of osteoclasts, cells which break down bone tissue, and increase the presence of osteoblasts which promote bulging bone formation [29,30,31], causing pain to the patient. Therefore, it is necessary to look for alternative therapies in order to modulate TME so as to increase the effectiveness of the existing antitumor agents used at present, as well as to reduce their side-effects. In this sense, as plant extracts have historically been utilized as medicinal remedies due to their anti-inflammatory, antioxidant and antimicrobial properties, several articles have been published showing the benefits of treating prostate cancer with natural compounds [32,33,34,35,36]. Indeed, many of those antitumor effects derive from TME modulation. For instance, resveratrol inhibits CAF differentiation, interrupts communication between tumor cells and TME and reduces TAM polarization to proinflammatory macrophages [37]. Likewise, cannabinoids impair CAF activation [38] and a compound containing Chinese herbs named Ligustilide induces their apoptosis and, therefore, reduces angiogenesis [39]. In addition, natural products are usually shown to be multi-targeted and less toxic than conventional therapies [40,41,42]. In fact, there is extensive literature supporting the application of natural bioactive compounds extracted from plants as adjuvants of current therapies in order to sensitize tumors and increase treatments’ efficacy. On the one hand, epigallocatechin-3-gallate (EGCG) (the main bioactive element of green tea), curcumin (obtained from turmeric) and resveratrol (extracted from grapes) are known to act as radiosensitizers increasing reactive oxygen species, boosting cell death and by inhibiting pro-tumoral pathways [43,44]. On the other hand, EGCG and glycyrrhizin (the major active constituent of licorice) have been shown to increase the effect of many different chemotherapeutic agents by enhancing their bioavailability, by interacting with receptors and by modulating chemoresistance-related proteins [43,44,45,46]. In addition, it has also been described that EGCG, glycyrrhizin and eugenol (present in cinnamon) can improve ADT by interacting with androgen receptors and enzymes [45,47]. Thus, innovative adjuvant strategies need to be developed to improve conventional therapies against cancer by adding adjuvant natural bioactive elements. In this regard, a natural nutritional mixture named Ocoxin has shown antitumor properties by itself against different primary and metastatic cancers where it increased apoptosis, caused a cell-cycle arrest and modulated TME by reducing CAF and TAM migration to the stroma [48,49,50,51]. Moreover, Ocoxin sensitized tumor cells to chemotherapy and reverted the pro-tumoral effect and chemoresistance caused by CAFs [48,49,50]. These effects of a nutritional mixture arise as a result of the combination of the ingredients that compose Ocoxin, such as green tea, cinnamon, licorice and vitamins, among others [47,52,53,54,55]. Furthermore, Ocoxin has been administered in clinics in order to mitigate the side-effects provoked by standard anticancer therapies, showing promising results in oncological patients, including those suffering from prostate cancer [56,57,58,59]. Bearing this in mind, we aimed to study whether Ocoxin could be used as a complement of the currently administered antitumor therapies Docetaxel, Enzalutamide and Olaparib.

## 2. Materials and Methods

### 2.1. Cell Lines

Three different prostate-cancer cell lines were analyzed in this study: human 22Rv1 and LNCaP cells (ATCC, Manassas, VA, USA) and murine RM-1 cells (ATCC, Manassas, VA, USA). Murine RM-1 cells were cultured in DMEM medium and human 22Rv1 and LNCaP cells were grown in RPMI-1640 medium (Gibco, Waltham, MA, USA), all of them supplemented with 10% Fetal Bovine Serum (FBS) (Gibco, Waltham, MA, USA) and 1% of Antibiotic–Antimycotic solution (Gibco, Waltham, MA, USA). Moreover, human primary osteoblasts (P10971) and prostate-tumor-associated fibroblasts (HC-6223) (Innoprot, Derio, Spain) were cultured in specific osteoblast and fibroblast growth medium (Innoprot, Derio, Spain) in order to obtain their secretomes. All cell lines were cultured at 37 °C in a humidified atmosphere in the presence of 5% CO_2_.

### 2.2. Ocoxin

Ocoxin is a nutritional mixture containing natural ingredients such as plant extracts, amino acids, vitamins and minerals (Table 1).

### 2.3. Cell-Viability Assay

Several cell-viability assays were performed with all the three prostate-cancer cell lines in order to study the cytotoxic effect of Ocoxin alone or combined with chemotherapy. First, 5 × 10^4^ cells/mL were cultured in 96-well plates in complete medium for 24 h in the case of RM-1 and 22Rv1 cells and 72 h for LNCaP. Then, cells were treated with different dilutions of Ocoxin ranging from 1:500 to 1:50 (V/V_f_) (Catalysis S.L., Toledo, Spain), Docetaxel (2.5–12 nM), Enzalutamide (12.5–50 µM) and Olaparib (2.5–10 µM) (Selleckchem, Houston, TX, USA) for 48 and 72 h in 1% FBS supplemented medium. Cell viability was measured by using PrestoBlue™ Cell Viability Reagent (Invitrogen, Waltham, MA, USA) for 2 h following manufacturer’s indications so as to assess the most effective doses. Afterwards, the effect of combinations of Docetaxel, Enzalutamide and Olaparib with Ocoxin were tested following the same protocol.

### 2.4. Cell-Cycle Analysis

Cell cycle was analyzed in 22Rv1, LNCaP and RM-1 cells treated with Ocoxin. First, 1.5 × 10^5^ cells/mL were cultured in 6-well plates under standard conditions, and then they were treated with the 1:100 and 1:50 dilutions of Ocoxin for 48 h in 1% FBS supplemented medium. Afterwards, cells were trypsinized, washed with PBS, and fixed with 70% ethanol for 30 min at 4 °C. Finally, cells were again washed and incubated with the FxCycle^TM^ PI/RNase Staining Solution (Invitrogen, Waltham, MA, USA) following the manufacturer’s instructions. Changes in cell cycle were analyzed by flow cytometry using the Gallios cytometer (Beckman Coulter, Brea, CA, USA).

### 2.5. mRNA Sequencing for LNCaP Cell Gene-Expression Analysis

In order to study molecular changes promoted by Ocoxin in human prostate-cancer cells, an mRNA sequencing was carried out. To begin with, LNCaP cells were cultured in 6-well plates under standard conditions for 72 h at a concentration of 1.5 × 10^5^ cells/mL. Afterwards, cells were treated with 1:50 Ocoxin dilution in 1% FBS-supplemented medium for 48 h. Then, detached cells were discarded through PBS washing and the RNA of adhered cells was isolated with the Total RNA purification kit (Norgen, Thorold, ON, Canada). Three sample replicates were extracted for each treatment. In order to perform the mRNA sequencing, first, the quantity and quality of the RNA was evaluated using the Qubit^TM^ RNA HS Assay Kit (Invitrogen, Waltham, MA, USA) and Agilent RNA 600 NanoChips (Agilent Technologies, Santa Clara, CA, USA). After, sequencing libraries were prepared using the “TruSeq^®^ Stranded mRNA Library Prep” kit, TruSeq^®^ RNA Single Indexes and TruSeq^®^ RNA CD Index Plate (Illumina, San Diego, CA, USA). Later, starting from 1 µg of total RNA, mRNA was purified, fragmented and primed for cDNA synthesis with the SuperScript^TM^ II Reverse Transcriptase (Invitrogen, Waltham, MA, USA) for 10 min at 25 °C, 15 min at 42 °C, and 15 min at 70 °C, and finished at 4 °C. The second cDNA strand was synthesized with Illumina reagents at 16 °C for 1 h, then A-tailing and adaptor ligation were performed and enrichment of libraries was achieved by PCR (30 s at 98 °C; 15 cycles of 10 s at 98 °C; 30 s at 60 °C; 20 s at 72 °C; 5 min at 72 °C and pause at 4 °C). Finally, libraries were visualized on an Agilent 2100 Bioanalyzer using the Agilent High Sensitivity DNA kit (Agilent, Santa Clara, CA, USA) and quantified using Qubit^TM^ dsDNA HS DNA kit (Invitrogen, Waltham, MA, USA).

### 2.6. Transcriptomic Analysis of LNCaP Cells Treated with Ocoxin

After obtaining mRNAseq results, data were analyzed in order to understand in which processes Ocoxin is involved. All the analyses were based on the Kyoto Encyclopedia of Genes and Genomes (KEGG) database [55]. First, all the significantly deregulated pathways were identified including all the altered genes. Afterwards, the most significantly deregulated genes were classified according to their differential expression, upregulated or downregulated, and the pathways they were involved in were analyzed using the KEGG mapper tool [60].

### 2.7. Quantification of the Differential Expression of Genes Altered by Ocoxin through RT-qPCR

Based on the data obtained from the mRNAseq, the differential expression of several genes involved in the cell cycle (KEGG ID: hsa04110) and protein processing in endoplasmic reticulum (KEGG ID: hsa04141), which is implicated in the reticulum stress and therefore in cell death, was analyzed by RT-qPCR in LNCaP cells which had been untreated or treated with 1:50 of Ocoxin. First, mRNA was purified as described above and 1 µg of RNA was retrotranscribed into cDNA using iScript cDNA Synthesis Kit (Bio-Rad, Hercules, CA, USA). Afterwards, the RT-qPCR was carried out using specific primers for the selected genes (Table 2) and SYBR Green as a fluorophore. Finally, relative expression of each gene was normalized to the internal control gene actin-β with the ΔΔCt method.

### 2.8. Obtention of Cancer-Associated Fibroblast- and Osteoblast-Derived Secretomes

Secretomes or conditioned media (CM) of human osteoblasts and prostate-tumor-associated fibroblasts were obtained after culturing 2 × 10^5^ cells/mL in 24-well plates with their specific growth medium. After 24 h, old medium was replaced for fresh medium and secretomes were collected 24 h later. Finally, all the obtained CM was centrifuged for 5 min at 4000 rpm and stored at −20 °C.

### 2.9. Chemoresistance Analysis in Prostate-Cancer Cells in the Presence of CAF and Osteoblast Secretomes

Tumor cells were cultured in RPMI medium supplemented with 1% FBS for 24 h and then fresh medium was added diluted 1:2 with CAF’s or osteoblast’s CM for another 24 h. Then, cells were treated with the 12.5 nM dose of Docetaxel in combination with the 1:50 dilution of Ocoxin for 48 h with regular culture medium and cell viability was analyzed using PrestoBlue™ Cell Viability Reagent (Invitrogen, Waltham, MA, USA).

### 2.10. Cell Migration Assay

On the one hand, migration of 22Rv1 cells was studied through the wound healing assay. To achieve this, 2 × 10^5^ cells/mL were cultured on 24-well plates under standard conditions until 90% confluence was achieved. Afterwards, cells were treated with 10 µg/mL of Mitomycin C (Sigma-Aldrich, Saint Louis, MO, USA) for 2 h in 1% FBS-supplemented medium in order to stop cell division. Then, a scratch was created in every well. After washing, either fresh medium supplemented with 1% FBS (as a control) or CAF- or osteoblast-secretomes diluted 1:2 in fresh medium was added to the plates for 48 h in the presence or absence of the 1:50 dilution of Ocoxin. Finally, the percentage of the scratch covered by the cells was quantified with the ImageJ software (version 1.53t).

On the other hand, the migratory capacity of LNCaP cells was evaluated using Transwell inserts (Corning Inc., Corning, NY, USA). Cells were cultured on the top of the inserts with standard medium and were let adhere to the membrane for 3 h. Then, the medium of the lower compartment was changed for fresh medium supplemented with 1% FBS or for either CAF- or osteoblast-secretomes diluted 1:2 in fresh medium and the same concentration of Ocoxin was added. Cells were let migrate for 48 h and, subsequently, they were fixed in 3.7–4% formaldehyde (PanReac AppliChem, Castellar del Vallès, Spain) and stained with 0.4% Cristal Violet (Sigma-Aldrich, Saint Louis, MO, USA). Lastly, insert membranes were mounted for the microscopic analyses.

### 2.11. Animals

In vivo experiments were carried out with 6–8 week male C57BL/6J mice (Janvier Labs, Le Genest-Saint-Isle, France). Animals were maintained in line with institutional guidelines and national and international laws for experimental animal care, and all the experimental procedures were approved by the Ethical Committee of the University of the Basque Country (CEID) and by institutional, national and international guidelines regarding the protection and care of animals used for scientific purposes (Reference M20-2022-076).

### 2.12. In Vivo Prostate-Cancer Tumor Development

RM-1 murine prostate-cancer cells were diluted in PBS at a concentration of 1.5 × 10^6^ cells/mL and 100 µL were subcutaneously injected in the right flank of mice. Then, animals were randomly divided into 4 groups of 7 mice so as to start the different treatments on the following day. Control group received a vehicle solution. The second group of mice was treated with an oral dose of Ocoxin (100 µL) daily, the third one received intraperitoneal injections of 5 mg/kg bodyweight of Docetaxel every other day and, finally, the fourth group received the combination of both treatments, that is, a daily dose of Ocoxin and an intraperitoneal injection of Docetaxel on alternate days. After 12 days of treatment, mice were sacrificed and tumors were either frozen in O.C.T^TM^ Compound (Sakura Finetek, Alphen aan den Rijn, The Netherlands) or fixed in 3.7–4% formaldehyde (AppliChem, Darmstadt, Germany) for 18 h at 4 °C and embedded in paraffin for histological analyses.

### 2.13. Immunohistochemical and Immunofluorescence Analyses

Samples were fixed for 10 min in ice-cold acetone (PanReac AppliChem, Castellar del Vallès, Spain) and permeabilized with PBS-Triton 0.05%. Afterwards, non-specific proteins were blocked by incubating the samples with PBS 5% FBS for 1 h and, then, tissues were incubated with specific antibodies overnight at 4 °C. The following antibodies were used: anti-caspase-3 (1:200) and anti-Ki67 (1:200) (purchased from Abcam, Cambridge, UK). Then, sections were washed with PBS three times and the secondary antibody Alexa Fluor^®^ 488 conjugated goat anti-rabbit IgG H&L (1:2000) (Abcam, Cambridge, UK) was added for 1 h. Finally, sections were again washed and slides were mounted with a DAPI containing mounting medium (Abcam, Cambridge, UK). Differences in angiogenesis were analyzed in vivo with immunohistochemical procedures. To do so, 5 µm thick slides were obtained from paraffin-embedded tissues, and after paraffin removal through an alcohol gradient, an antigen retrieval step was performed by incubating the samples in citrate buffer at 96 °C for 30 min. Then, endogenous peroxidase was blocked by adding 3% of H_2_O_2_ in PBS. Afterwards, non-specific proteins were blocked by incubating the samples with 3% FBS in PBS for 1 h and, then, tissues were incubated with the specific anti-CD31 (1:100) (Abcam, Cambridge, UK) antibody overnight at 4 °C. Then, sections were washed with PBS three times and the secondary anti-rabbit IgG H&L (Abcam, Cambridge, UK) was added for 1 h. Finally, sections were again washed, counterstained with eosin for 5 s and dehydrated and mounted.

Immunofluorescence samples were examined under the Zeiss Axioskop fluorescence microscope (Zeiss, Oberkochen, Germany) and the immunohistochemistry slides were observed under the Olympus BX50 optic microscope (Olympus Soft Imaging Solutions, Hamburg, Germany). Finally, expression levels were quantified through the ImageJ software (National Institutes of Health, Bethesda, MD, USA) and results were expressed as the mean expression of at least X tumor sections per treatment.

### 2.14. Statistical Analysis

The statistical analyses of all the experiments, except the mRNAseq, were performed using GraphPad Prism 5.0 (GraphPad software Inc., San Diego, CA, USA). Every in vitro experiment was carried out at least three times and the in vivo assay was performed twice. To begin with, the Kolmogorov–Smirnov normality test was run in all the experiments. When normal distribution was assumed, the One-Way ANOVA was used with the Bonferroni’s post-hoc test and for non-parametric tests the Kruskal–Wallis test followed by Dunnett’s post-hoc test was applied. In every graph, data are expressed as the mean value (±standard deviation (SD)).

Regarding the mRNAseq, the multiExperiment Viewer version 4.9.0 (J. Craig Venter Institute, Rockville, MD, USA) was utilized for the statistical analyses and, afterwards, the One Way ANOVA the false discovery rate (FDR) correction was applied.

## 3. Results

### 3.1. Ocoxin Altered the Viability and Cell Cycle of Human Prostate-Cancer Cell Lines

**Figure 1:**
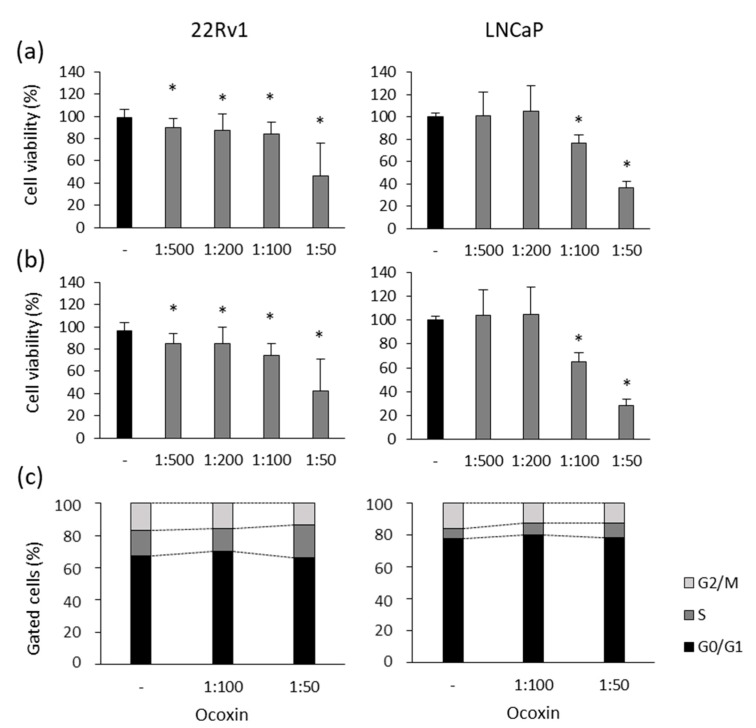
Cytotoxic effect of Ocoxin on the viability and cell cycle of human prostate-cancer cells. (**a**,**b**) Cell viability was quantified using the Presto Blue^TM^ Cell Viability Reagent after treating 22Rv1 and LNCaP cells with 1:500, 1:200, 1:100 and 1:50 dilutions of Ocoxin for (**a**) 48 and (**b**) 72 h. Cell viability was affected in a dose-dependent manner. (**c**) The proportion of cells gathered in each cell-cycle phase was measured using flow cytometry using the FxCycle^TM^ PI/RNase Staining Solution after the treatment with the 1:100 and 1:50 dilutions of Ocoxin for 48 h. A cell-cycle arrest in the S phase and a decrease in cell number was observed in G2/M phase. Differences versus untreated cells were considered statistically significant at *p* < 0.05 (*) according to the Kruskal–Wallis test.

First of all, the viability of human 22Rv1 and LNCaP prostate-cancer cells was analyzed in the presence of different doses of Ocoxin. As shown in Figure 1, the nutritional supplement reduced cell viability by more than 50% in both cell lines when treated with the highest dose at 48 (Figure 1a) and 72 h (Figure 1b). Moreover, the 1:100 dilution also reduced cell viability in 22Rv1 cells by 25% and by 35% in LNCaP cells at 72 h (Figure 1b).

To elucidate if the reduction in cell viability caused by Ocoxin is due to a cell-cycle arrest, changes in the cell cycle were studied after the exposure of prostate-cancer cells to the natural compound for 48 h. Figure 1c shows that Ocoxin caused a gathering of cells in the S phase with the subsequent decrease in cells in the following G2/M phase from 17% to 14% in 22Rv1 and from 16% to 12% in LNCaP cells with the highest dose of Ocoxin.

### 3.2. mRNAseq for the Analysis of Gene Expression of LNCaP Cells Treated with Ocoxin

In order to gain insights into the mechanism of action of Ocoxin, changes in the gene expression of LNCaP cells were analyzed through mRNAseq after treating them with the 1:50 dilution of Ocoxin for 48 h. According to the results, a total number of 23.726 genes were altered by the treatment, but only 614 genes were significantly differentially expressed after FDR correction; precisely, 173 were downregulated and 441 were upregulated. Considering all the deregulated genes, analyses based on the KEGG database showed that Ocoxin altered the expression of genes that are involved in three main pathways: cell cycle, DNA replication and p53 signaling (Table 3).

**Figure 2:**
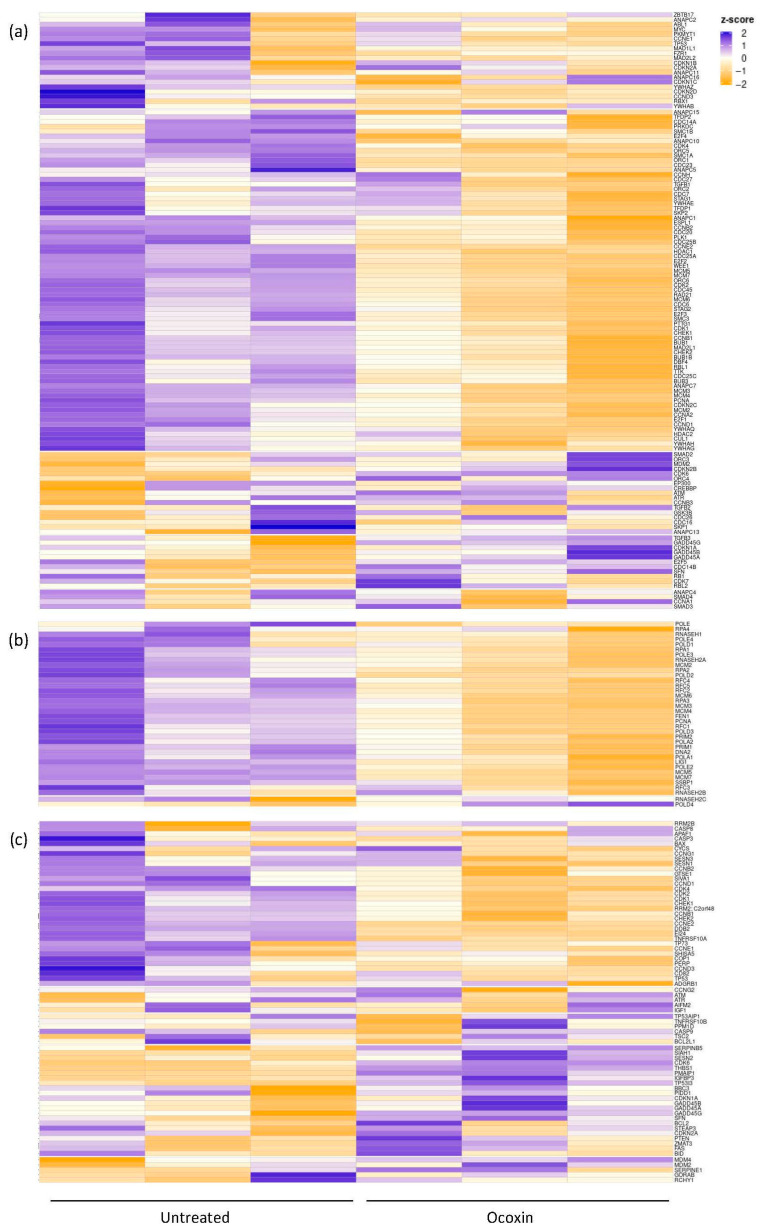
Heatmap of the differential expression of genes involved in the cell cycle, p53 signaling pathway and DNA replication in prostate-cancer cells treated with Ocoxin. LNCaP cells were treated for 48 h with the 1:50 dilution of Ocoxin. mRNA expression levels were measured through mRNAseq in both treated and untreated cells. (**a**) Cell cycle, (**b**) DNA replication and (**c**) p53 signaling pathway.

The differential expression of the genes is displayed in Figure 2, where Ocoxin is shown to either increase or decrease the expression of genes involved in the cell-cycle (Figure 2a) and p53 signaling pathway (Figure 2c) and downregulate the genes involved DNA replication (Figure 2b). In addition, an in-depth analysis was performed focused only on the significantly deregulated genes according to their expression pattern (upregulated or downregulated). As shown in Table 4, even though Ocoxin caused the overexpression and infraexpression of genes involved in metabolic and cancer-related processes and in the p53 pathway, most of the upregulated genes were related, as well, to the PI3K-Akt, mTOR and MAPK signaling pathways, protein processing in the endoplasmic reticulum, autophagy and apoptosis, and the downregulated genes were principally implicated in cell cycle and DNA replication, among others (Table 4).

### 3.3. Analysis of the Differential Expression of LNCaP Cell Genes Treated with Ocoxin by RT-qPCR

**Figure 3:**
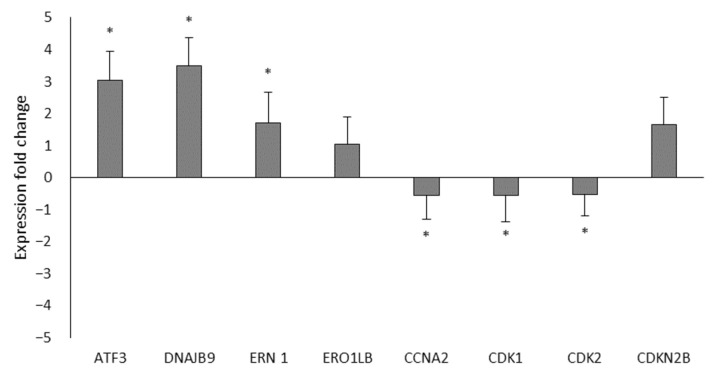
Differential expression of the mRNAseq through RT-qPCR. The differential expression of eight genes was analyzed by RT-qPCR between LNCaP cells that were untreated or treated with the 1:50 dose of Ocoxin. The genes involved in the protein processing in the endoplasmic reticulum (ATF3, DNAJB9, ERN1 and ERO1LB) were upregulated and those from the cell cycle were downregulated (CCNA2, CDK1 and CDK2) except CDKN2B, which was upregulated. Differences versus untreated cells were considered statistically significant at *p* < 0.05 (*) according to the One-Way ANOVA.

The differential expression of eight genes implicated in the cell cycle and in the protein processing in the endoplasmic reticulum that was shown to be altered by Ocoxin according to the mRNAseq results was quantified, by RT-qPCR, in LNCaP cells. As seen in Figure 3 and in accordance with the mRNAseq, on the one hand, among the genes involved in the protein processing in the endoplasmic reticulum ERN1, ERO1LB, DNAJB9 and ATF3 were all upregulated. On the other hand, also in line with the results obtained in the mRNAseq, the genes of the cell cycle, CDK1, CDK2 and CCNA2 were downregulated and DCKN2B was upregulated.

### 3.4. Ocoxin as an Adjuvant Agent of Docetaxel, Enzalutamide and Olaparib Increased the Cytotoxic Effect in Human Prostate-Cancer Cell Lines

**Figure 4:**
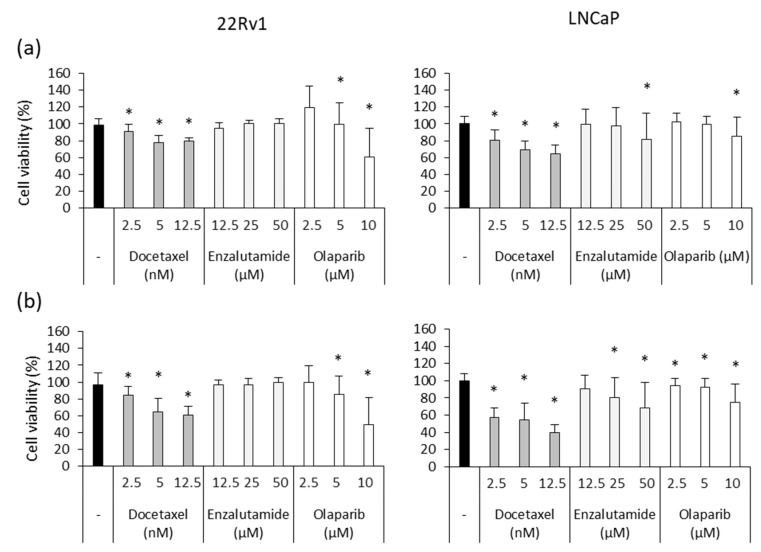
Cytotoxic effect of chemotherapeutic agents on human prostate-cancer cells. Different doses of Docetaxel (2.5 nM, 5 nM, 12.5 nM), Enzalutamide (12.5 µM, 25 µM, 50 µM) and Olaparib (2.5 µM, 5 µM, 10 µM) were added to 22Rv1 and LNCaP cells in order to assess their cytotoxic capacity after (**a**) 48 and (**b**) 72 h. Docetaxel and Olaparib exerted a dose-dependent effect in both cell lines while 22Rv1 was resistant to Enzalutamide and only the highest doses affected LNCaP cells. Differences versus untreated cells were considered statistically significant at *p* < 0.05 (*) according to the Kruskal–Wallis test.

To analyze the adjuvant effect of Ocoxin, we first studied the cytotoxicity of routinely administered chemotherapeutic agents in prostate-cancer patients Docetaxel, Enzalutamide and Olaparib. Although both cell lines were sensitive to Docetaxel and Olaparib, 22Rv1 cells did not respond to Enzalutamide and LNCaP cells were only affected by the highest doses after 48 (Figure 4a) and 72 h (Figure 4b). Focusing on the highest concentrations of chemotherapy, while the 12.5 nM dose of Docetaxel reduced 22Rv1 and LNCaP cell viability by 40% and 60%, respectively, after 72 h, 10 µM of Olaparib decreased cell number by 60% in the 22Rv1 cell line and 25% in LNCaP cells (Figure 4b).

**Figure 5:**
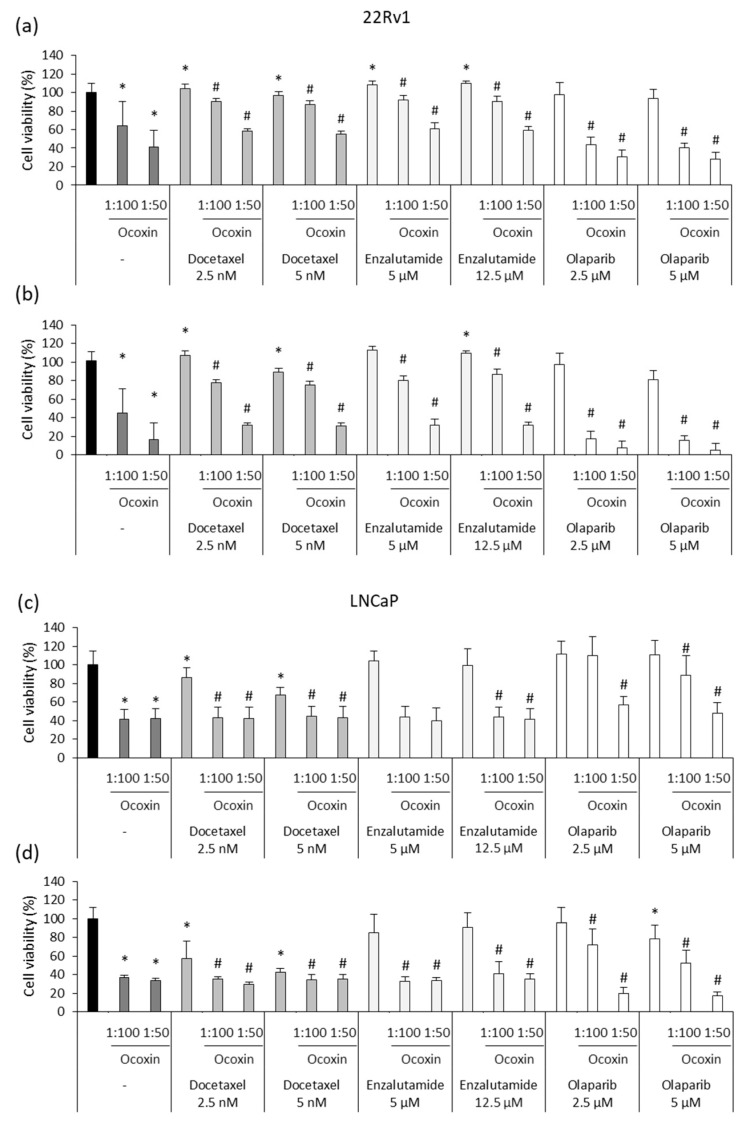
Cytotoxic effect of chemotherapeutic agents combined with Ocoxin on 22Rv1 and LNCaP human prostate-cancer cells. Different doses of Docetaxel (2.5 nM, 5 nM), Enzalutamide (5 µM, 12.5 µM) and Olaparib (2.5 µM, 5 µM) were added to cells combined with Ocoxin (1:100, 1:50). (**a**) 22Rv1 cells treated for 48 h, (**b**) 22Rv1 cells treated for 72 h, (**c**) LNCaP cells treated for 48 h, (**d**) LNCaP cells treated for 72 h. All the combined treatments reduced cell viability more than chemotherapy alone. Differences were considered statistically significant at *p* < 0.05 according to the Kruskal–Wallis test when comparing untreated cells versus cells treated with chemotherapy alone (*) and when comparing chemotherapy alone versus combinations with Ocoxin (#).

Later, the antitumor activity of Ocoxin was analyzed in combination with chemotherapy. In detail, two doses of Docetaxel, Enzalutamide or Olaparib were combined with the 1:50 and 1:100 dilutions of Ocoxin for 48 and 72 h and cell viability was measured. Results showed that the adjuvant treatment reduced cell viability more than chemotherapy alone in every case (Figure 5). In 22Rv1 cells, while chemotherapy alone barely affected cell viability, the combination of the lowest doses of Docetaxel, Enzalutamide or Olaparib with Ocoxin 1:50 decreased cell viability by up to 42%, 39% and 69% at 48 h, respectively (Figure 5a), by up to 68% with Docetaxel and Enzalutamide, and by 93% with Olaparib at 72 h (Figure 5b). Likewise, in LNCaP cells, the combination of 2.5 nM of Docetaxel with the 1:50 dilution of the nutritional supplement diminished the number of viable cells from 13% to 57% at 48 h and also decreased cell viability combined with the 5 µM of Enzalutamide and 2.5 µM of Olaparib by 60% and 43%, respectively, while both drugs did not affect cells on their own at all (Figure 5c). This effect was enhanced at 72 h, where the 1:50 dilution of Ocoxin reduced cell viability by 70%, 67% and 80% when combined with the lowest concentrations of Docetaxel, Enzalutamide and Olaparib, respectively (Figure 5d).

### 3.5. Ocoxin Reduced the Pro-Migratory Effect and Chemoresistance Produced by Soluble Factors Derived from Osteoblasts and CAFs Secreted on Human Prostate-Cancer Cells

**Figure 6:**
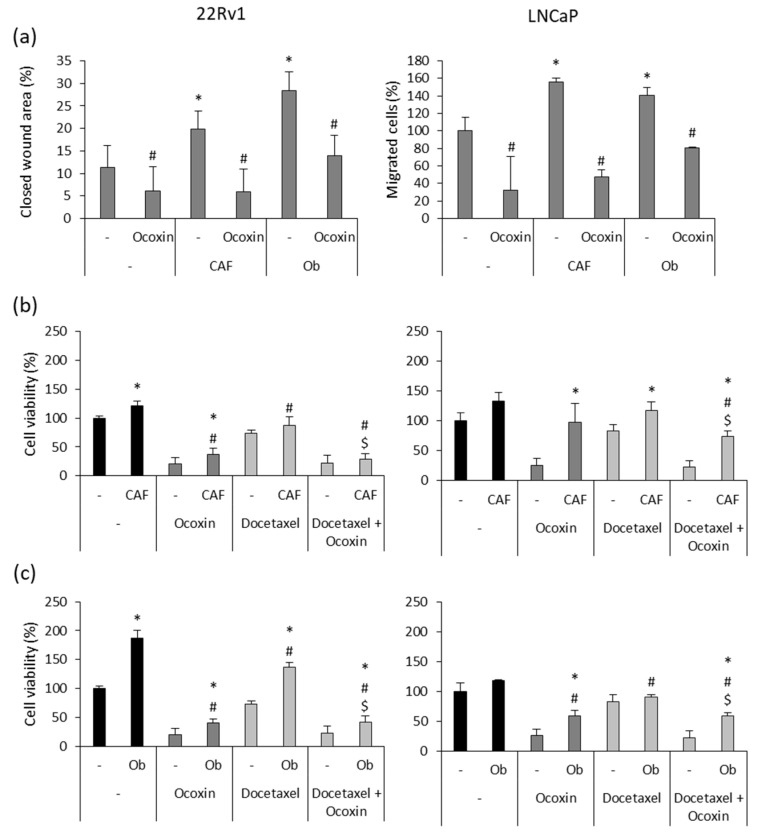
Effect of Ocoxin on the promigratory capacity and chemoresistance conferred by soluble factors derived from cancer-associated fibroblasts (CAFs) and osteoblasts (Obs) to 22Rv1 and LNCaP prostate-cancer cells. (**a**) Cell migration was determined after treating them with the 1:50 dilution of Ocoxin in the presence CAF- and Ob-derived conditioned medium (CM) for 48 h. Although both CMs increased the migratory capacity of prostate-cancer cells, Ocoxin reverted cell migration. Differences between treatments were considered statistically significant at *p* < 0.05 according to the Kruskal–Wallis test when comparing cells cultured under normal conditions versus those cultured with CM (*) and when comparing each control to the treatment with Ocoxin (#). (**b**,**c**) Chemoresistance was determined in after the treatment with 12.5 nM of Docetaxel alone or combined with the 1:50 dilution of Ocoxin in the presence of CM collected from (**b**) CAFs and (**c**) Obs for 48 h. Both CMs reduced the efficacy of the treatments compared to those added under standard conditions. However, the adjuvant therapy reduced cell viability more than Docetaxel alone. Differences between treatments were considered statistically significant at *p* < 0.05 according to the Kruskal–Wallis test when comparing treatments under normal conditions versus treatments in the presence of CM (*), between the control and the treatments being both with CM (#) and between Docetaxel alone versus the combination being both in the presence of CMs ($).

The main concern regarding prostate cancer is the development of metastasis. One of the cellular mechanisms involved in this process is cell migration. Hence, since TME components provide soluble factors to prompt tumor growth and invasion, we analyzed the effect of CAF and osteoblast CM on the migratory capacity of cancer cells. Furthermore, considering that Ocoxin impaired the support provided by CM to cancer cells, we analyzed the effect of the natural mixture on cell migration in CM-stimulated cells. As shown in Figure 5a, both CAF and osteoblast CMs enhanced the migratory capacity of prostate-cancer cells. However, Ocoxin reverted the pro-migratory effect caused by CMs, reducing CAF and osteoblast CM-stimulated cell migration by around 70% and 50%, respectively, in both cell lines, reaching the migration levels that cells showed when not in the presence of CMs. Moreover, it is known that TME confers chemoresistance to tumors. Thus, bearing in mind that the cytotoxic effect of the combination of Docetaxel and Ocoxin was higher than chemotherapy alone, we studied if the adjuvant treatment could also revert chemoresistance in prostate-cancer cells exposed to CM derived from CAFs and osteoblasts. Figure 6b,c shows that CM increased cell viability in both cell lines regardless of the treatment. In particular, both CMs were shown to enhance cell viability more in 22Rv1 cells than in LNCaP. The most remarkable difference was observed in 22Rv1 cells, whereas osteoblast CM almost duplicated cell viability after 48 h (Figure 6c), CAF CM only enhanced it by 20% (Figure 6b). In addition, the combination of Docetaxel and Ocoxin reduced cell viability more than Docetaxel alone in the presence of the soluble factors derived from either CAFs or osteoblasts in both prostate-cancer cell lines. While the chemotherapeutic agent alone reduced cell viability by around 25% in every case, the combination increased cell death by up to 75% in 22Rv1 cells in the presence of either CAF- or osteoblast-derived soluble factors (Figure 6b,c) and up to 20% (Figure 6b) and 50% (Figure 6c) in LNCaP cells when they were stimulated with CAF or osteoblast CM, respectively.

### 3.6. Ocoxin Altered the Viability and Cell Cycle of a Murine Prostate-Cancer Cell Line

**Figure 7:**
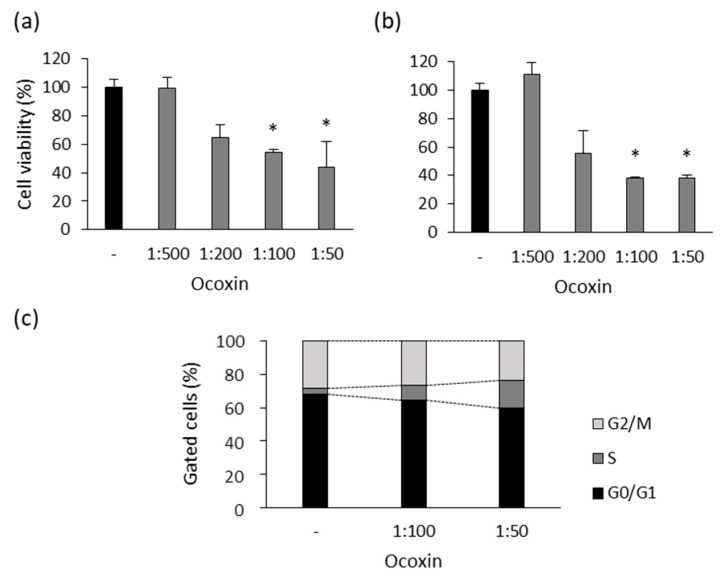
Effect of Ocoxin on the viability and cell cycle of murine prostate-cancer cells. RM-1 cells were treated with 1:500, 1:200, 1:100 and 1:50 dilutions of Ocoxin for (**a**) 48 and (**b**) 72 h and cell viability was quantified. Ocoxin affected cell viability in a dose-dependent manner. (**c**) RM-1 cells were treated with the 1:50 dilution of Ocoxin for 48 h and the percentage of cells gathered in each cell-cycle phase was measured. Ocoxin caused a cell-cycle arrest in the S phase and decreased the number of cells in G2/M and G0/G1 phases. Differences versus untreated cells were considered statistically significant at *p* < 0.05 (*) according to the Kruskal–Wallis test.

Since Ocoxin affected the viability of human prostate-cancer cells, on the one hand, we analyzed the effect of the compound in the murine RM-1 cell line. Several doses of Ocoxin ranging from 1:500 to 1:50 were used to treat the cells for 48 and 72 h. As shown in Figure 7, Ocoxin reduced cell viability by around 50% with the 1:100 and 1:50 dilutions at 48 h (Figure 7a) and by approximately 60% at 72 h (Figure 7b). On the other hand, cell-cycle analyses revealed that Ocoxin increased the number of cells gathered in the S from 4% to 9% and 17% with the 1:100 and 1:50 doses, respectively, causing a cell-cycle arrest which reduced the number of cells in phase G2/M from 28% to 26% and 24% with both dilutions (Figure 7c).

### 3.7. Ocoxin as an Adjuvant Agent of Docetaxel Increased the Cytotoxic Effect in a Murine Prostate-Cancer Cell Line

**Figure 8:**
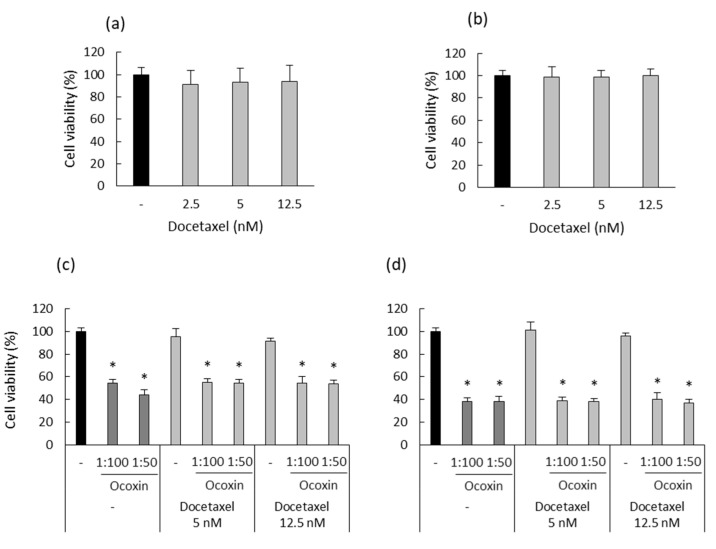
Cytotoxic effect of Docetaxel alone and combined with Ocoxin on murine prostate-cancer cells. (**a**,**b**) Different doses of Docetaxel (2.5 nM, 5 nM, 12.5 nM) were added to RM-1 cells in order to assess their cytotoxic capacity at (**a**) 48 and (**b**) 72 h. This cell line was shown to be resistant to Docetaxel. (**c**,**d**) RM-1 cells were treated with Docetaxel (12.5 nM, 5 nM) combined with Ocoxin (1:100, 1:50) for (**c**) 48 and (**d**) 72 h in order to study the cytotoxic effect of the adjuvant therapy. All the combined treatments reduced cell viability. Differences were considered statistically significant at *p* < 0.05 (*) according to the Kruskal–Wallis test when comparing chemotherapy alone with combinations with Ocoxin.

After testing the anticancer properties of Ocoxin as an adjuvant therapy on human prostate-cancer cells in vitro, we also analyzed whether Ocoxin exerted the same effect in murine RM-1 prostate-cancer cells. First of all, the cytotoxic effect of different concentrations of Docetaxel (2.5, 5 and 12.5 nM) was analyzed for 48 and 72 h in order to choose an optimum dose to treat RM-1 cells with the combination of Docetaxel and Ocoxin afterwards. Figure 8a,b show that Docetaxel alone did not cause any cytotoxic effect against RM-1 cells in the analyzed conditions. Nevertheless, although RM-1 cells were shown to be resistant to Docetaxel, the combination with both the 1:50 or the 1:100 dilutions of Ocoxin reduced cell viability significantly from approximately 5% to 50% at 48 h (Figure 8c) and to 60% at 72 h, respectively (Figure 8d).

### 3.8. The Administration of Ocoxin as an Adjuvant of Docetaxel Reduced Prostate Tumor Volume In Vivo by Increasing Apoptosis and by Decreasing Proliferation and Angiogenesis

**Figure 9:**
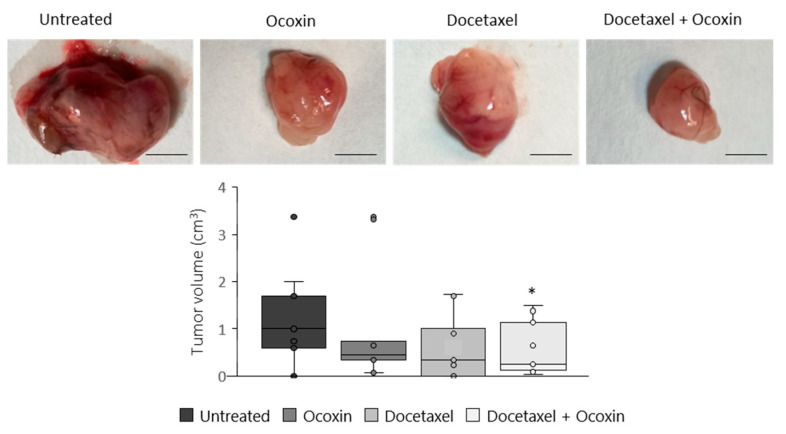
Antitumor effect of Ocoxin as an adjuvant of Docetaxel in mice with prostate cancer. Mice bearing subcutaneous prostate-cancer tumors were treated with a daily oral dose of 100 µL of Ocoxin and with 5 mg/Kg bodyweight of Docetaxel intraperitoneally every other day (alone or in combination) for 12 days. Then, tumors were extracted and volume was measured. The adjuvant therapy reduced tumor volume significantly compared to that of untreated mice. The scale bar corresponds to 5 mm length. Differences were considered statistically significant at *p* < 0.05 (*) according to the one-way ANOVA.

**Figure 10:**
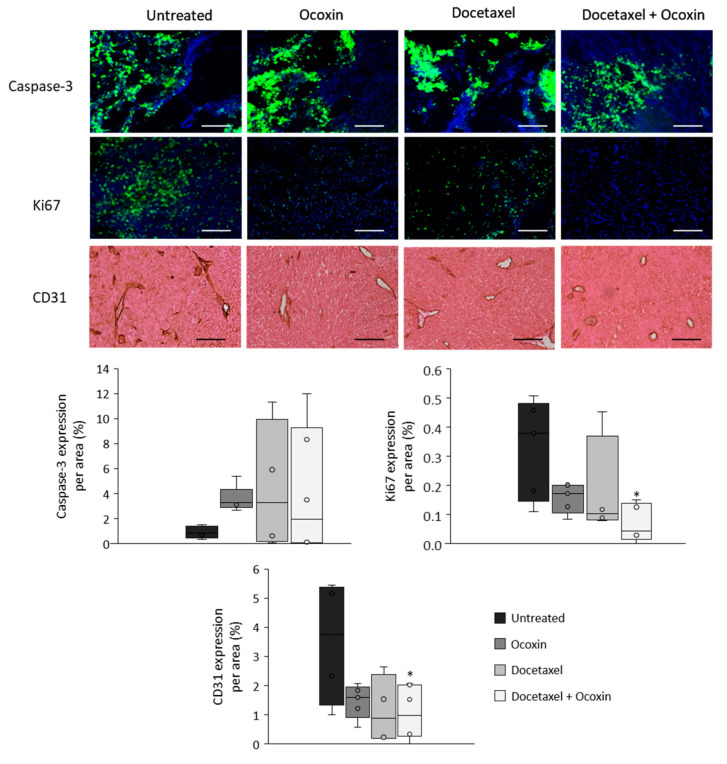
Apoptosis, proliferation and angiogenesis in tumors of mice bearing prostate cancer treated with Docetaxel and Ocoxin. Analyses of apoptosis (caspase-3) and proliferation (Ki67) rates were performed through immunofluorescence and angiogenesis (CD31) levels were determined by immunochemistry in tumors obtained from mice with prostate cancer that had been treated with a daily dose of 100 µL of Ocoxin and with 5 mg/kg bodyweight of Docetaxel intraperitoneally every other day (alone or in combination) for 12 days. All the three treatments showed a trend towards an increase in apoptosis and the combination of Docetaxel and Ocoxin reduced cell proliferation and angiogenesis significantly compared to untreated mice. The scale bar corresponds to 250 µm length. Differences were considered statistically significant at *p* < 0.05 (*) compared to tumors of untreated mice according to the One-Way ANOVA.

Based on the in vitro results, we tested the antitumor capacity of the adjuvant treatment in vivo. To do so, C57BL/6J mice with subcutaneous prostate-cancer tumors were treated with Docetaxel and Ocoxin on their own or combined. Although bodyweight in the mice was unchanged, while Docetaxel reduced tumor volume by 44% compared to untreated mice, the combination of Docetaxel and Ocoxin decreased it by 64% (Figure 9). Therefore, in order to gain insights into the reason leading to the reduction in tumor size and also to confirm our in vitro results, tumor-cell proliferation and apoptosis analyses were performed using immunofluorescence. As shown in Figure 10, the supplementation of the chemotherapeutic agent with Ocoxin caused an increase in the anti-proliferative effect of Docetaxel, that is, the adjuvant therapy decreased tumor-cell proliferation by approximately 60% compared to Docetaxel alone. However, although all the three treatments increased apoptosis within tumors, no significant differences could be observed between groups due to the high variability in data. Finally, angiogenesis levels were quantified inside tumors using the endothelial cell marker CD31. Figure 10 shows that all the three treatments reduced angiogenesis, although only the value of the combined therapy was significant, which reduced angiogenesis by 70% compared to the untreated mice.

## 4. Discussion

Prostate cancer is one of the leading causes of cancer-related deaths among men in the world [1]. While primary tumors are curable by surgery or radiotherapy, the treatment of metastatic prostate cancer is still a challenge. Even though Docetaxel, Enzalutamide or Olaparib increase life expectancy, only one-third of the patients survive for more than 5 years and their QoL is usually deteriorated as a consequence of undesired side-effects caused by chemotherapy [2,11,61]. Hence, new strategies are being explored including the supplementation of current medicines with natural neoadjuvant agents. On this matter, many authors have summarized the chemopreventive and anticancer attributes of Chinese herbal medicines and plant-derived bioactive compounds in prostate cancer [34,36,62,63]. Furthermore, phytochemicals have been tried in vitro and in vivo combined with chemotherapy to improve treatment outcome showing beneficial responses [64,65,66,67,68]. Additionally, natural supplements have also been dispensed in clinics to mitigate side effects and to help the recovery of patients under treatment against different cancers, which, in addition, have been demonstrated to increase survival rates [46,69,70]. However, results are not always consistent and the mechanisms of action of the combinations have not been deeply studied.

In this work, we tested whether a nutritional mixture named Ocoxin could serve as a novel adjuvant agent to improve chemotherapy in prostate cancer. This compound contains green tea, licorice and cinnamon extract among other natural elements, which have shown to exert antioxidant, anti-inflammatory, immunoregulatory and antitumoral activities when administered alone or as a mixture in vitro and in vivo in various types of cancer [48,49,50,51]. Moreover, Ocoxin enhanced life expectancy and relieved side effects caused by the current therapies in patients [56,57,58,59]. Interestingly, a clinical study with prostate-cancer subjects receiving radiotherapy and/or chemotherapy was performed, where Ocoxin was employed as a supportive treatment; this showed a significant improvement in patients’ QoL, a better response to chemotherapy and an increase in overall survival [71]. Thereby, we delved into the underlying anticancer processes in which Ocoxin actively participates. To start with, we studied the effect of the mixture against human and murine prostate-cancer cells. Consistent with our previous findings, Ocoxin reduced prostate-cancer cell viability in a dose-dependent way [48,50,51]. Many studies report the cytotoxic capacity of plant extracts obtained from green tea, licorice root or cinnamon among others, which are included in the composition of Ocoxin [52,53,72]. Since this effect could lie in distinct mechanisms, we investigated if cell-viability decrease was as a result of a cell-cycle arrest. As expected, the natural supplement caused a delay in the cell-cycle progression in all the three tested cell lines, 22Rv1, LNCaP and RM-1. More specifically, Ocoxin caused the accumulation of prostate-cancer cells in the S phase, whereas previous reports showed that the natural compound halted cell cycles in the Sub G1 phase in colorectal cancer and in G0/G1 in melanoma [48,51]. Actually, these findings are gathered in a review confirming that Ocoxin acted at different cell-cycle points depending on the type of cancer [73]. In this regard, we found out that the effect produced by Ocoxin on the cell cycle of prostate-cancer cells is mediated by the modulation of gene expression. Notably, most of the genes downregulated by Ocoxin were mainly related with the cell cycle and cell division, such as cyclin-dependent kinases (CDKs) such as CDK1 and CDK2, among others.

CDKs are essential proteins to progress the cell cycle. In particular, CDK1 and CDK2 modulate the expression of transcription factors in order to alter the gene expression of different elements across the cell-cycle phases. Gao et al., reported that Oridonin contributes to the inhibition of gastric-cancer cell growth by the downregulation of CDK1 and the induction of cell-cycle arrest in the G2/M phase [74]. Moreover, cyclin A2 (CCNA2), which was also downregulated by Ocoxin, activates CDK2 to drive the transition from the S phase to the M phase, blocking the passage of cells to the mitotic process [75]. Li et al., reported that the overexpression of the CCNA2–CDK2 complex is associated with the occurrence of several cancers, namely, lung cancer, stomach cancer, leukemia, breast cancer and other tumors [76]. Thus, CCNA2–CDK2-complex inhibitors are being analyzed as antitumor therapies. According to this, a component isolated from traditional Chinese medicine, *Salvia miltiorrhiza* Bunge, Tanshinone IIA, provoked cell-cycle arrest and apoptosis and inhibited the proliferation of lung adenocarcinoma cells through the downregulation of the CCNA2-CDK2 complex, the same effect observed using Ocoxin [77]. Furthermore, cyclin-dependent kinase inhibitor 2B (CDKN2B), highly overexpressed by Ocoxin, is known to form a complex with CDK4 and CDK6, which prevents the activation of the CDKs. On this point, Xia et al., reported the strength of CDKN2B as a tumor suppressor by inhibiting cell cycle and glycolysis [78] and Zhang and his collaborators correlated the overexpression of CDKN2B with apoptosis in hepatocellular carcinoma cells treated with Veramapil + Doxorubicine [79]. Hence, the downregulation of the CCNA2–CDK2 complex and the overexpression of CDKN2B produced by Ocoxin could have mediated not only the gathering of the cells in the S phase of the cycle but also the induction of tumor cells to an apoptotic stage. In fact, our preliminary in vitro results confirmed that human prostate-cancer apoptosis was increased two-fold after the treatment with the nutritional mixture. In addition, even though prostate-cancer cells were rather resistant to chemotherapy, the supplementation with Ocoxin diminished cell viability more than the drugs (Docetaxel, Enzalutamide and Olaparib) administered alone. In line with this, many works support that some of the ingredients present in Ocoxin, such as green tea and licorice, enhanced chemosensitivity in prostate-cancer cells [43,45,80]. Remarkably, EGCG and quercetin have shown to sensitize prostate-cancer cells to Docetaxel and Enzalutamide, among others, by improving their anti-proliferative effect and by inhibiting androgen receptor signaling [67,68]. Hence, Ocoxin could have also sensitized cancer cells to chemotherapy owing to the properties of those plants.

In addition, in consonance with our in vitro results, Ocoxin as adjuvant therapy reduced tumor volume significantly in vivo, more than Docetaxel alone. However, although an increasing tendency of apoptosis was observed in murine tumors, no significant differences was detected between treatments, probably due to the variability observed within the animal groups. Nevertheless, it is important to note that cells can be induced to apoptosis in different ways, including intrinsic mitochondrial pathways, extrinsic death receptor pathways and perforin/granzyme pathways, all of them starting with the cleavage of caspases and finishing with DNA fragmentation [81]. In this regard, reticulum stress-mediated apoptosis is widely described [81,82,83]. Endoplasmic reticulum (ER) can cause the activation of the unfolded protein response (UPR) under stressful situations, a process that takes part in the re-establishment of the cellular homeostasis protecting cells from stress. After prolonged ER stress stimuli, UPR is activated and cells are induced to death. In this respect, proteins such as the activating transcription factor-6 (ATF6), inositol-requiring enzyme-1 (IRE1) and protein kinase RNA-activated (PKR)-like ER kinase (PERK) act as UPR sensor proteins which activate three different signaling pathways leading cells to the induction of autophagy or apoptosis. Interestingly, Ocoxin upregulated the expression of several genes related to the ER stress process, including the DnaJ heat shock protein family (Hsp40) member B9 (DNAJB9), endoplasmic reticulum oxidoreductase 1 beta (ERO1B), activating transcription factor 3 (ATF3), endoplasmic reticulum to nucleus signaling 1 (ERN1), DNA damage inducible transcript 3 (DDIT3), tribbles pseudokinase 3 (TRIB3) and protein phosphatase 1 regulatory subunit 15A (PPP1R15A), among others.

It is described that the activation of ATF3 promotes apoptosis and cell-cycle arrest by inhibiting the ubiquitination of the Murine Double Minute Clone 2 (MDM2), a p53 tumor suppressor regulator [84,85]. Moreover, Muñoz-Guardiola and his colleagues reported that the alpha-hydroxylated polyunsaturated fatty acid (ABTL0812) promotes the overexpression of DDIT3 causing tumor-cell apoptosis and that TRIB3 overexpression inhibited the protein kinase B (Akt) and the mammalian target of rapamycin (mTOR) complex 1 (AKT-MTORC1) pathway provoking cell death by autophagy [86]. Nowadays, the induction of cells to this self-degradative process has emerged as a new alternative to kill cancer cells; thus, new anticancer therapeutic strategies try to impulse tumor-cell autophagy with pharmacological compounds [87]. On this matter, natural compounds, namely, salinomycin, resveratrol and tetrahydrocannabinol, are known to activate cancer-cell death by autophagy-dependent mechanisms through the ER stress response [88,89,90]. Ocoxin also upregulated several genes, which are directly linked to the ER stress response, which leads us to speculate that the antitumor action of Ocoxin could also be mediated by the induction of cancer-cell death via ER stress response promoting apoptosis or autophagy. Nonetheless, further analyses will be performed in order to confirm this and to delve into the antitumor mechanism of Ocoxin. In addition, the implication of TME during prostate-cancer development and progression has to be considered when designing novel therapeutic drugs. As previously described, prostate cancer causes metastasis mainly in bones, where diverse molecules and cells including CAFs and osteoblasts play a pivotal role. CAFs secrete cytokines and growth factors that prompt tumor-cell growth and boost colonization [15]. Meanwhile, osteoblasts interact with the different signals received from cancer cells and from the TME, which enables tumor invasion by remodeling bones and by promoting the development of metastasis [30,91]. Curiously, prostate-cancer cells show a preference for osteoblast-rich areas [92], suggesting that osteoblasts assist metastatic development.

In this study, we confirmed that CAFs and osteoblasts increased prostate-cancer cell viability and migration in vitro, an effect which was reverted by Ocoxin. In fact, we formerly corroborated that Ocoxin also reduced in vitro the migration capacity of fibroblasts themselves. Along with that, recent studies discuss the effect of natural compounds, not only in prostate-cancer cells but and also in CAFs and osteoblasts. For instance, Pietrovito et al., discovered that cannabinoids act on cancer cells and CAFs at the same time by inhibiting CAF activation [38]; Silk and her colleagues reported that resveratrol downregulates Transforming Growth Factor β (TGFβ) expression while it exerts an anti-proliferative and a pro-apoptotic effect in prostate CAFs [37]; and Ma and her collaborators found out that a Chinese natural medicine is able to cause CAF death and to reduce their pro-angiogenic capacity by reducing Vascular Endothelial Growth Factor A (VEGFA) production [39,93]. However, we could not detect any alteration regarding the presence of CAFs and TAMs in vivo due to the small number of infiltrated cells in the tumor stroma. This result could be a consequence of the limitations of the experimental model, that is, the modest size of the tumors and the fact that it was subcutaneous development, which caused tumor encapsulation and hindered external cell infiltration. Still, we observed that Ocoxin alone reduced blood-vessel formation in vivo, and that the combination of Ocoxin and Docetaxel decreased angiogenesis significantly compared to untreated mice, which confirms that Ocoxin could also mediate, in part, the antitumor capacity by the modulation of the elements that compose TME. Moreover, in this work, we also showed that even though the presence of CAF- and osteoblast-derived soluble factors reduced the cytotoxic capacity of Docetaxel, the adjuvant therapy decreased cell survival significantly in human prostate-cancer cells more than chemotherapy alone. In concordance, we previously reported that Ocoxin reduces CAF-mediated chemoresistance and the pro-tumoral activity of metastatic melanoma [48] and we described the capacity of Ocoxin to cut down the infiltration of activated fibroblasts and macrophages into the tumor stroma in vivo as well [49,51]. Thus, taking everything into consideration, Ocoxin arises as a suitable supplement to be administered together with the current first- and second-line treatments in prostate cancer to reinforce chemotherapy in two ways, acting directly against tumor cells and modulating the TME.

## Figures and Tables

**Table 1:** Composition of Ocoxin per vial of 60 mL.

**Amino Acids**	
L-Glycine	1200 mg
L-Arginine	384 mg
L-Cysteine	122.4 mg
**Minerals**	
Zinc sulfate	48 mg
Glucosamine sulfate potassium chloride	1200 mg
Manganese sulfate	2.4 mg
**Plant Extracts**	
Licorice extract (*Glycyrrhiza glabra*)	120 mg
Green tea extract (*Canellia sinensis*)	15 mg
Cinnamon extract (*Cinnamomum verum*)	1.8 mg
**Vitamins**	
Vitamin C	72 mg
Vitamin B5	7.2 mg
Vitamin B6	2.4 mg
Vitamin B9	240 µg
Vitamin B12	1.2 µg

**Table 2:** Sequences of the primers used to validate gene expression by RT-qPCR.

Gene	Sequence
ATF3	Forward: AGAAAGAGTCGGAGAAGC
	Reverse: TGAAGGTTGAGCATGTATATC
DNAJB9	Forward: TGCAGAAGCATATGAAACAC
	Reverse: ACTAGTAAAAGCACTGTGTC
ERO1LB	Forward: GGAGGAATTCCGATTACATTTC
	Reverse: TTCCCCATAATCTGCATTTG
CDK1	Forward: ATGAGGTAGTAACACTCTGG
	Reverse: CCTATACTCCAAATGCAACTG
CDK2	Forward: TGTTATCGCAAATGCTGC
	Reverse: TCAAGAAGGCTATCAGAGTC
CCNA2	Forward: AGTATCATGGTGTTTCTCTCC
	Reverse: AATTTGTACTTGGCCACAAC
CDKN2B	Forward: GACTAGTGGAGAAGGTGC
	Reverse: TCATCATGACCTGGATCG
ERN1	Forward: GAATAGAAAAGGAATCCCTGG
	Reverse: TTCTTATTTCTCATGGCTCG

**Table 3:** The most significantly deregulated pathways according to the analysis based on KEGG database in human prostate-cancer cells treated with Ocoxin.

KEGG ID	Description
hsa04110	Cell cycle
hsa03030	DNA replication
hsa04115	p53 signaling pathway

**Table 4:** Summary of the KEGG pathways including the highest number of significantly altered genes classified into upregulated and downregulated.

**UPREGULATED**		
**KEGG ID**	**Description**	**Genes**
hsa01100	Metabolic pathways	50
hsa05022	Pathways of neurodegeneration	15
hsa04151	PI3K-Akt signaling pathway	13
hsa05200	Pathways in cancer	13
hsa05208	Chemical carcinogenesis—reactive oxygen species	12
hsa05014	Amyotrophic lateral sclerosis	10
hsa01240	Biosynthesis of cofactors	10
hsa05010	Alzheimer disease	9
hsa04141	Protein processing in endoplasmic reticulum	9
hsa05168	Herpes simplex virus 1 infection	9
hsa05165	Human papillomavirus infection	8
hsa04510	Focal adhesion	8
hsa04144	Endocytosis	8
hsa05225	Hepatocellular carcinoma	8
hsa04140	Autophagy	7
hsa04210	Apoptosis	7
hsa04150	mTOR signaling pathway	7
hsa04115	p53 signaling pathway	7
hsa04010	MAPK signaling pathway	7
hsa05016	Huntington disease	7
**DOWNREGULATED**		
**KEGG ID**	**Description**	**Genes**
hsa04110	Cell cycle	18
hsa03030	DNA replication	10
hsa05200	Pathways in cancer	8
hsa01100	Metabolic pathways	8
hsa05166	Human T-cell leukemia virus 1 infection	6
hsa04218	Cellular senescence	6
hsa04914	Progesterone-mediated oocyte maturation	6
hsa04114	Oocyte meiosis	5
hsa05169	Epstein–Barr virus infection	5
hsa04115	p53 signaling pathway	4
hsa05207	Chemical carcinogenesis—receptor activation	4
hsa05203	Viral carcinogenesis	4
hsa04080	Neuroactive ligand-receptor interaction	4
hsa03460	Fanconi anemia pathway	4
hsa04611	Platelet activation	4
hsa03430	Mismatch repair	4
hsa05202	Transcriptional misregulation in cancer	4
hsa05161	Hepatitis B	4
hsa03440	Homologous recombination	4
hsa05165	Human papillomavirus infection	4

## Data Availability

Data is unavailable due to privacy.
